# Bioinformatic analysis of m6A “reader” YTH family in pan-cancer as a clinical prognosis biomarker

**DOI:** 10.1038/s41598-023-44143-1

**Published:** 2023-10-13

**Authors:** Lin Li, Chao Tang, Jianqing Ye, Da Xu, Chuanmin Chu, Lei Wang, Qiwei Zhou, Sishun Gan, Bing Liu

**Affiliations:** 1 Department of Urology, The Third Affiliated Hospital of Second Military Medical University, 700 North Moyu Road, Shanghai, 201805 China; 2 National Clinical Research Center for Child Health of the Children’s Hospital, Zhejiang University School of Medicine, No. 3333, Binsheng Road, Hangzhou, 310052 China; 3 Department of Urology, School of Medicine, Xinhua Hospital, Shanghai Jiaotong University, 1665 Kongjiang Road, Shanghai, 200092 China

**Keywords:** Biomarkers, Oncology

## Abstract

The m6A methylation of mRNA has been demonstrated to interact with the “Reader”. YTH domain family is one of the readers containing five members involved in the progression of multiple tumors. The present study aimed to explore the YTH family's role in seventeen cancer types. Data were downloaded from The Cancer Genome Atlas (TCGA) dataset and analyzed by Software R 3.6.3. Using different bioinformatics methods, including analyses of the overall survival (OS) and disease-free survival (DFS), Gene Set Variation Analysis (GSVA) enrichment. Genomics of Drug Sensitivity in Cancer (GDSC), CIBERSORT algorithm, multivariate and lasso cox regression analysis our results reveal that, while the expression of the YTH domain family varies distinctively in different cancer types the expression of YTH family is upregulated in most cancer types, especially in liver cancer, and the liver cancer prediction model established herein includes YTHDF1 and YTHDF2. Therefore, the results of the present study have demonstrated that the YTH domain family has the potential to predict the prognosis of cancer and the sensitivity to immunotherapy.

## Introduction

Among all RNA chemical modifications, m6A is the most common and most widely distributed epigenetic transcriptional modification in eukaryotic RNAs. Researchers have gradually discovered that m6A modifications have multiple biological functions. M6A affects various cellular processes, including cell differentiation, tumor formation, and regulation of circadian rhythms. Dysregulation of RNA methylation is closely associated with cancer development and progression^1^. N6-methyladenosine (m6A) modification has recently received much attention as the most common means of RNA methylation regulation and is expected to be a new target for anti-cancer therapy^2^

YTH (YT521-B homology family) gene family has five members, all of which encode reader proteins with m6A recognition function and are named after a highly conserved YTH structural domain^3^. Previous studies on YTH proteins have focused on animals, particularly mammals, and have classified mammalian YTH proteins into three major groups: YTHDC1 (YTH domain-containing protein 1), YTHDC2 (YTH domain-containing protein) and YTHDF (YTHdomain-containing family protein), of which YTHDF contains YTHDF1-3.

Proteins containing YTH structural domains are involved in many RNA processes, such as mRNA splicing, nuclear export, translation and attenuation of post-transcriptional regulation, by initially recognizing the m6A modification of target RNAs and subsequently directing different complexes to regulate RNA signaling pathways, including RNA folding, RNA splicing, protein translation, and RNA metabolism. Recently, proteins containing YTH structural domains were found to play an important role in post-transcriptional modifications that regulate gene expression associated with cancer and other processes including cell cycle progression and cell proliferation^4^. Recent evidence suggests that YTHDF1 acts as an oncogene by regulating different signaling pathways in various cancers, such as colorectal cancer (CRC)^5,6^, hepatocellular cancer^7^, and breast cancer^8^. Bai et al.^4^ reported that YTHDF1 mediated the Wnt/β-catenin pathway by interacting with the downstream targets WNT6 and FZD9 mRNA to affect the tumorigenicity and stem cell-like activity of CRC cells. In addition, YTHDF1 expression was significantly associated with various features of CRC such as depth, lymph node metastasis (LNM), tumor stage, and poor prognosis, suggesting that its expression may be a useful independent prognostic factor for CRC. Mechanistically, YTHDF1 can be directly upregulated by the oncogenic transcription factor c-Myc^6^. However, there is no systematic study on the expression and function of the YTH gene family in the pan-cancer context.

This study aims to further broaden the understanding about the role of the YTH gene family in the pan-cancer context through public databases (including Cancer Genome Atlas, "GSVA" R package, and GEPIA2 database), in an attempt to provide new perspectives for cancer diagnosis and treatment. In this study, we evaluated the expression, prognostic value, function and pathway enrichment of individual YTH gene family members in the pan-cancer setting and discusses the relevance of tumor immune cell infiltration to the YTH family.

## Methods

### Data collection and processing

Public gene expression data and clinical annotations were retrieved in the latest version of Cancer Genome Atlas (TCGA) database (updated in 2018)^9^. mRNA expression and clinical data, including tumor stage, histology subtype, gender and overall survival (OS), were obtained from TCGA database. The cancer proteome atlas (TCPA) database provides a tumor protein profile by integrating RPPA chip data from the TCGA project^10^. P values, hazard ratios (HR), and 95% confidence intervals (CI) were visualized using the 'forestplot' R package. Univariate and multivariate cox regression analyses were performed and a 1-, 2- and 3-year overall recurrence nomogram was drawn on the multivariate cox proportional hazards analysis through ‘rms’ R package.

### Gene set variation analysis (GSVA)

To examine the differences of YTH family genes in biological processes, we used "GSVA" R package to conduct a GSVA enrichment analysis^11^. The gene set "h.all.v7.2 " for GSVA analysis was downloaded from the MSigDB database.

### Correlation analysis

GEPIA2 (http://gepia.cancer-pku.cn/index.html) the “Correlation Analysis” module was used to analyze the correlation between YTH domain family and immune checkpoint molecules (PD-L1\PD-L2\CTLA4) and quantify the relative abundance of the key gene in (Epithelial-mesenchymal transition (EMT), WNT, apoptosis and NOTCH signal pathway in 17 cancer types^12^.

### Calculation of abundance of invading cells in the tumor microenvironment (TME)

We used CIBERSORT algorithm to quantify the relative abundance of 22 types of immune cells in CRC by using the following parameters: the input mixture matrix as the gene expression matrix and the input of gene signature reference for 22 immune cell types from Newman et al.^13^.

### Survival analysis

Based on the correlation between the expressions of YTH family genes and patient survival, survminer package was used to determine the cutoff point of survival information. The "surv-cutpoint" function was used to dichotomize the expression of YTH family genes, and all potential cutting points were repeatedly tested to find the maximum rank statistic, and then the patients were divided into the expression-high group and the expression-low group according to the maximum selected log-rank statistic. Survival curves for prognostic analysis were generated using the Kaplan–Meier method, and the log-rank test was used to determine the significance of the differences. Univariate Cox regression model was used to calculate the HR between YTH family genes. All statistical analyses were two-side and p < 0.05 was considered statistically significant.

### Pharmacy response of YTH family

To evaluating the target-therapeutic response of YTH domain family, we download the data from the largest publicly available pharmacogenomics database [the Genomics of Drug Sensitivity in Cancer (GDSC), https://www.cancerrxgene.org/]. We processed the prediction by R package ‘pRRophetic’. Ridge regression was used to estimate the half-maximal inhibitory concentration (IC50) of the samples. The duplicate gene expression was summed up as a mean value using the batch effect of battle and tissue type of all tissues.

### Receiver operating characteristic (ROC) curve

The least absolute shrinkage and selection operator (LASSO) regression algorithm and the step function were performed repeatedly until the optimal model was found. For Kaplan–Meier curves, p-values, and HR with 95% CI were generated by log-rank tests and univariate cox proportional hazards regression. All the analytic methods and R packages were implemented by R (foundation for statistical computing 2020) version 4.0.3. p value < 0.05 was considered statistically significant.

### Declarations

The study was approved by the Ethics Committee of The Third Affiliated Hospital of Second Military Medical University. An exempt of written informed consent was granted by the Ethics Committee of The Third Affiliated Hospital of Second Military Medical University as no human participants or patient materials were included.

## Results

### The expression of YTH domain family in the pan-cancer context

**Figure 1:**
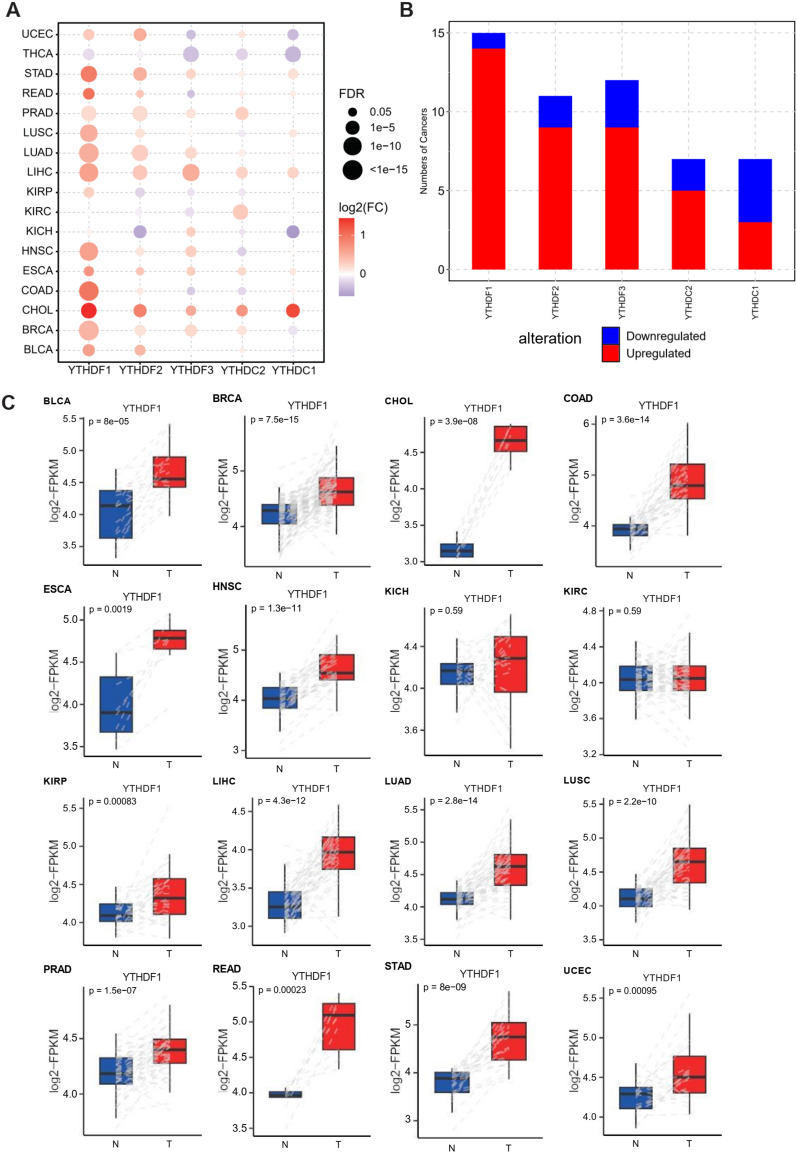
The expression of YTH domain family in pan-cancer. (**A**, **B**). Expression of YTH family gene in pan-cancer. The color of bubbles represents the difference in expression of YTH family genes between tumor tissues and paired normal para-cancer tissues. Red: up-regulated expression in tumor tissues; Blue, down-regulated in tumor tissue; Bubble size indicates significant difference. The height of the column indicates the number of tumor types involved in dysregulated expression of YTH family genes. (**C**) The expression of YTHDF1 in 16 cancer types. Red: tumor tissue; Blue: paired paracancer normal tissue.

To explore the “readers” involved in cancer progress, we searched the TCGA database and selected the pairwise correlative 17 tumors. The expression of RNA methylation YTH domain family member was calculated and evaluated (Fig. 1A,B). YTHDF1 was elevated in 14 of the 17 tumors but reduced in thyroid carcinoma (THCA), Kidney renal clear cell carcinoma (KIRC) and Kidney chromophobe (KICH). YTHDF2 expression was upregulated in 13 tumor cohorts in Uterine Corpus Endometrial Carcinoma (UCEC), Stomach adenocarcinoma (STAD), Lung adenocarcinoma (LUAD), Cholangio carcinoma (CHOL), Bladder Urothelial Carcinoma (BLCA) and Liver hepatocellular carcinoma (LIHC) as well. However, YTHDF3, YTHDC1 and YTHDC2 genetic alteration was various. YTHDF3 and YTHDC1 rose in 11 tumors. YTHDC2 was ascended in 9 cancers in lung squamous cell carcinoma (LUSC), esophageal carcinoma (ESCA), CHOL, LIHC and STAD. To clarify the differential expression in tumors, we further examined the YTH domain family and found that YTHDF1 was obviously elevated in rectum adenocarcinoma (READ), LIHC, CHOL and ESCA (Fig. 1C). In addition, the differential expression in protein levels of the YTH domain family was further verified in pan-cancer (Fig. S1).

### The clinical prognosis of YTH domain family in pan-cancer

**Figure 2:**
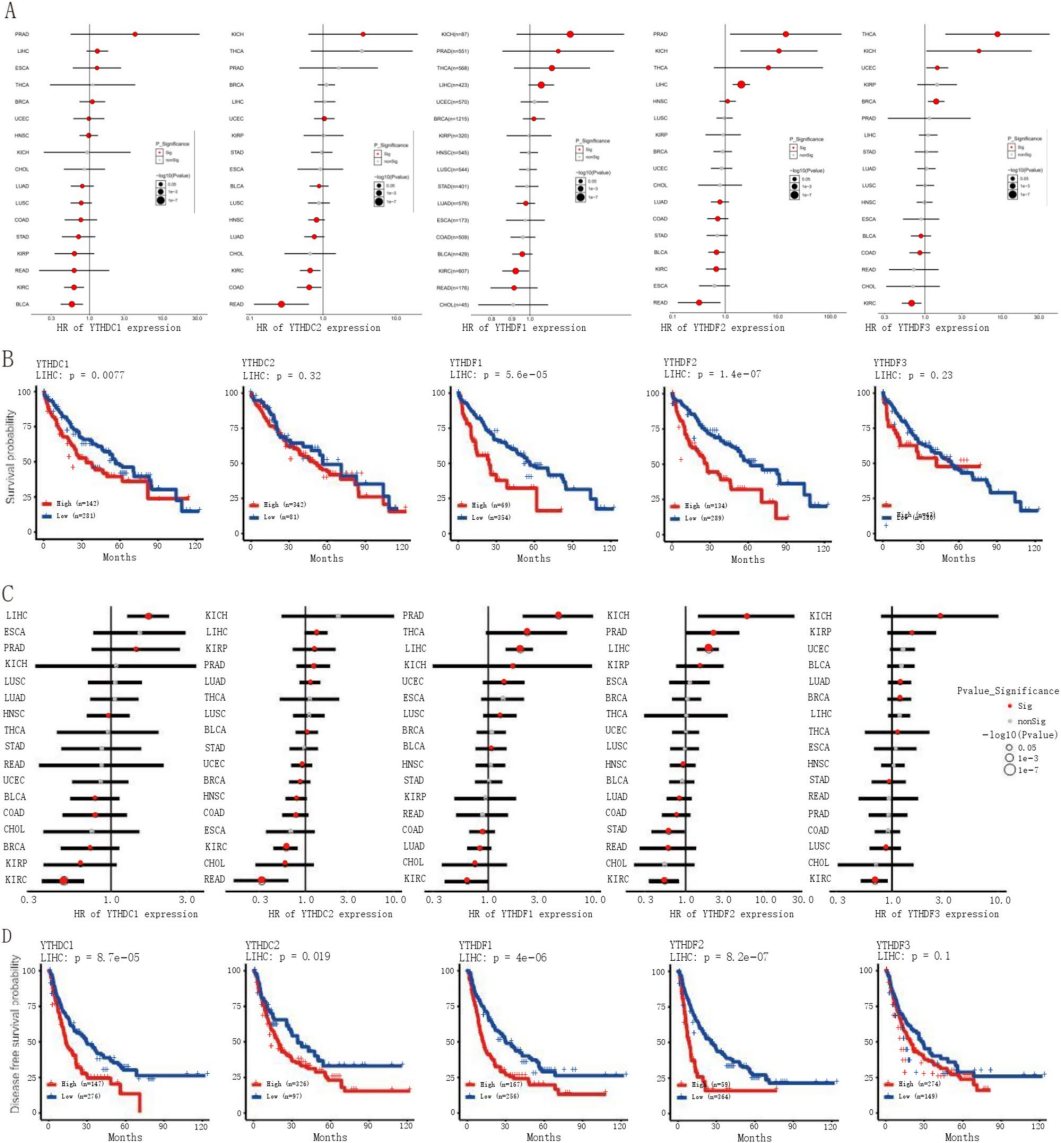
The prognosis and correlation analysis of YTH domain family. (**A**, **C**) The COX analysis between YTH domain family expression and the overall survival (**A**) and the progress free survival (**C**) of different cancer types. Red: YTH family expression significantly affects survival prognosis; Gray: No significant relationship between YTH family expression and survival prognosis. (**B**, **D**) the overall survival (**B**) and the progress free survival (**D**) of YTH domain family in LIHC. Red: High expression group of YTH family genes; Blue: Low expression group of YTH family gene.

To further explore the influence of YTH domain family in tumors, we firstly made a meta-analysis on OS differences of YTH domain family between high- and low-expression groups (Fig. 2A), and found that YTHDF1 upregulation was a risk factor associated with reduced OS in THCA, KICH, LIHC and UCEC, while it was a protective factor in BRCA, LUAD, BLCA, and READ. YTHDF2 expression also varied with tumors and was a risk factor in prostate adenocarcinoma (PRAD), KICH, THCA and LIHC, and a protective factor in colon adenocarcinoma (COAD), bladder urothelial carcinoma (BLCA), LUAD, KIRC and READ. YTHDF3 upregulation may reduce OS in KIRC and COAD, and may prolong in KICH, BRCA and THCA. Both YTHDC1 and YTHDC2 were detected as protective factors in KIRC, BLCA, CIAD and LUAD, and was a risk factor in different tumors. In addition to YTHDF3, YTH domain family genes were risk factors in LIHC. Consistently, the upregulation of YTHDF1/2 and YTHDC1/2 was associated with poor survival prognosis of LIHC patients, particularly for YTHDC1 and YTHDF1/2, which showed prognostic significance (Fig. 2B).

We further explored progress-free survival (PFS), and interestingly, we found that YTHDF1/2 was a risk factor for cancer progress in KICH, LIHC and PRAD, while YTHDF3 was a risk factor for cancer progress in KICH and KIRP, and a protective factor in KIRC and LUSC (Fig. 2C). In addition, YTHDC1 was found to promote cancer progress in LIHC and PRAD, and play a protective role in COAD, BRCA, KIRP and KIRC. YTHDC2 promoted PFS in KIRC, CHOL and READ, but promoted cancer progression in LIHC, KIRP, PRAD and LUAD (Fig. 2D).

### Enrichment analysis of YTH domain family in pan-cancer

**Figure 3:**
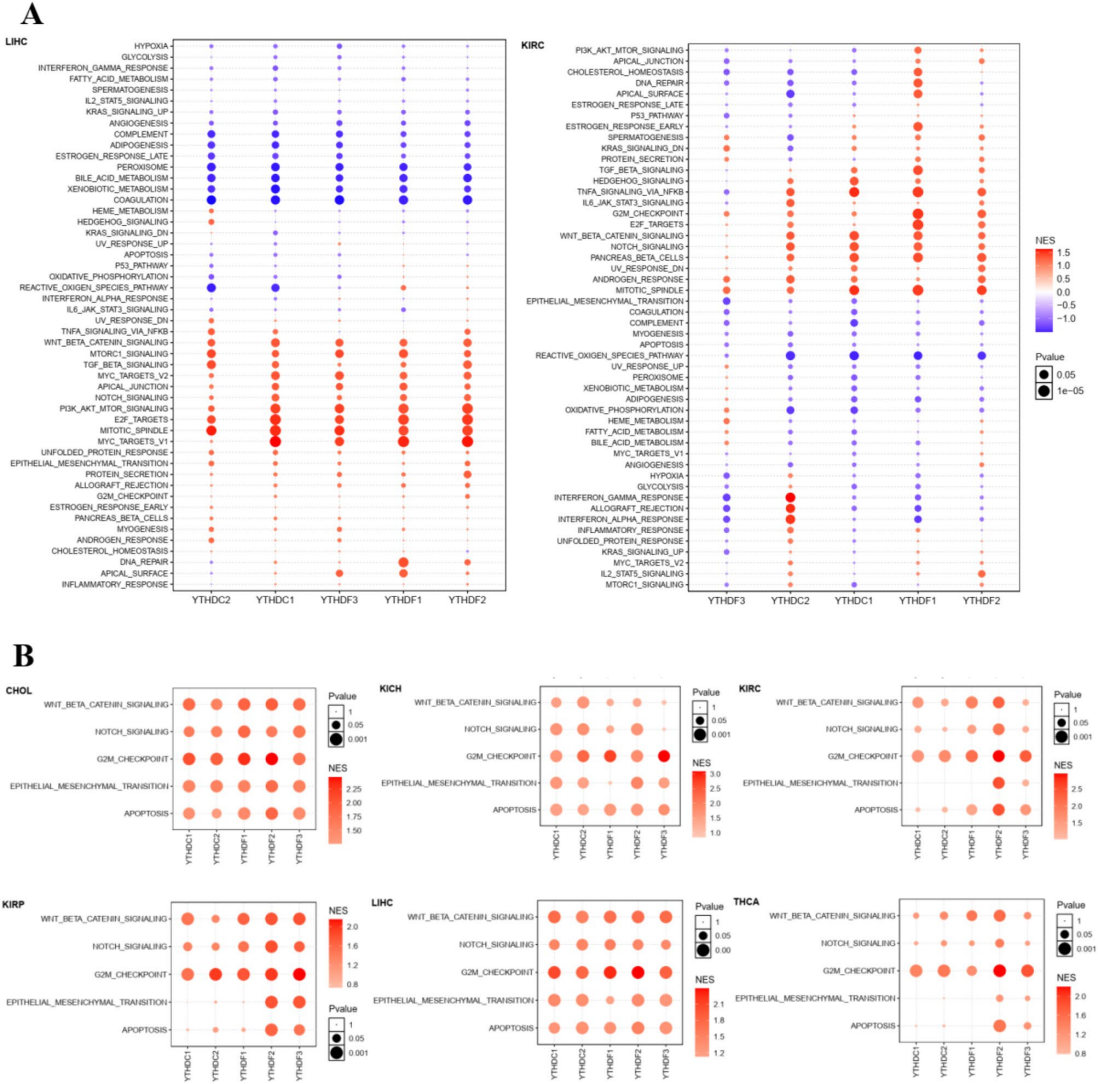
The enrichment analysis of YTH domain family. (**A**). Enrichment score (NES) of signal pathways significantly associated with YTH family expression in LIHC and KIRC; (**B**). Relationship between expression of YTH family and enrichment score of five signaling pathways (WNT, notch, cell cycling, EMT and apoptosis). Red: YTH family expression is related to signal pathway activation; Blue: YTH family expression is associated with signaling pathway inhibition. Bubble color indicates the enrichment fraction of signal pathway. Bubble size indicates the significance of NES.

GSEA enrichment analysis was performed for gene diseases significantly related to the expression of YTH family in 17 cancer types. An overview of the data demonstrated that signaling underwent various changes in different cancer types (Fig. S2), including LIHC and KIRC. It was found that multiple pathways were upregulated with high expression of YTH in LIHC and CHOL (Fig. 3), including WNT, TGF-β, NOTCH, PI3K/AKT/mTOR and MYC signaling pathways in LIHC, and YTH domain family also initiated the NOTCH, EMT, MYC signaling and WNT signaling in CHOL.

**Figure 4:**
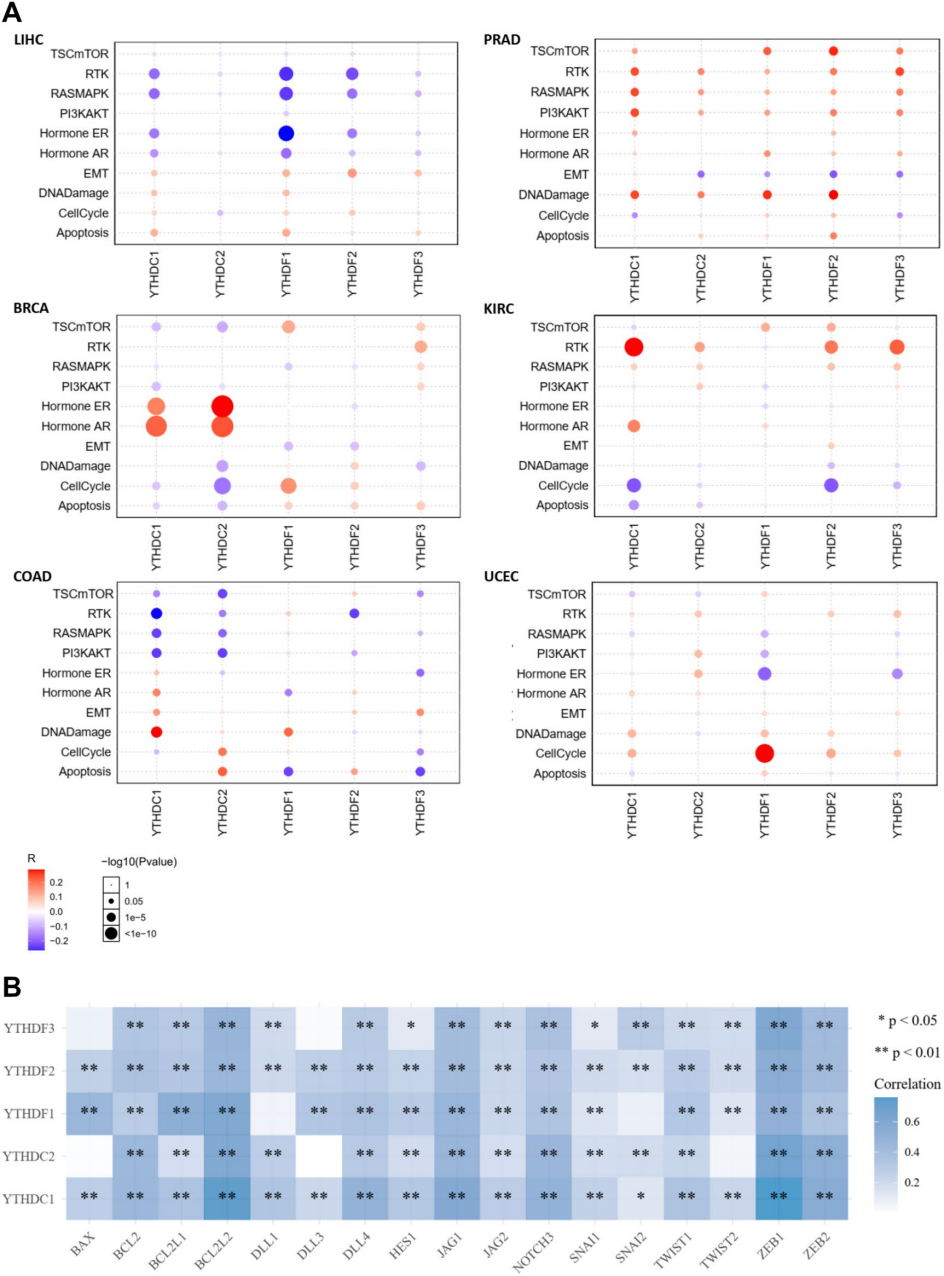
The correlation between related pathway protein expression and YTH domain family in LIHC, PRAD, BRCA, KIRC, COAD and UCEC. The protein expression of signaling pathway activated or inhibited by YTH family using TCGA-TCPA chip data in LIHC, PRAD, BRCA, KIRC, COAD and UCEC. The pathway included mTOR, RTK, RAS/MAPK, PI3K/AKT, ER, AR, EMT, DNA damage, Cell cycling and apoptosis. Red: the signal pathway is activated; Blue: the signaling pathway inhibited. Bubble color indicates the enrichment fraction of signal pathway. Bubble size indicates the significance of R.

To study the protein expression, we counted the data from TCPA database (http://www.tcpaportal.org) and found that YTH family was associated with diverse protein expressions. In LICH, the GSEA had low scores in RTK and RAS/MAPK, but was upregulated in EMT-related protein. The protein expression of KIRC, KIRP and BLCA was also high in RTK. It was found that cell cycle was arrested significantly in UCEC and STAD, and PI3K/AKT was upregulated in THCA (Fig. 4A).

Enrichment analysis of tumor-related signaling pathways at the protein level using TCGA-RPPA chip data from the TCPA database revealed a correlation between the activation or suppression of signaling pathways and the expression of YTH domain family genes in most tumors (Figs. 4A, S3). For instance, the expression of YTHDF1/2 and YTHDC1 genes in LIHC was negatively correlated with the activation of RTK, RAS/MAPK and ER hormone signaling pathways, and positively correlated with the activation of EMT, DNA damage, cell cycle and apoptosis signaling pathways. Moreover, KIRC, KIRP and BLCA also had high expression of RTK. Cell cycle was arrested significantly in UCEC and STAD, and PI3K/AKT was upregulated in THCA (Fig. 4A).

We tested the key genes in EMT, WNT, apoptosis and NOTCH signal pathway. For example, NOTCH signal of YTH domain family showed a strong positive correlation with DLL4, JAG1 and NOTCH3 in LIHC by Spearman correlation analysis, with the R value between 0.4 and 0.6. We also calculated the Bcl-2 family in Bcl-2, Bcl-xl, Bcl-w and bim with R value between 0.4 and 0.6. Assessment of EMT associated genes showed that both ZEB1 and ZEB2 were completely associated with YTH domain family, while SNAIL1, SNAIL2, TWIST1 and TWSIT2 showed weak correlations. Our meta-analysis on EMT signal on ZEB1 and ZEB2 showed a positive relation between ZEB1 and YTH domain family, especially on YTHDC1 and YTHDC2 (Fig. 4B).

### The relationship between YTH domain family and immune cell infiltration

**Figure 5:**
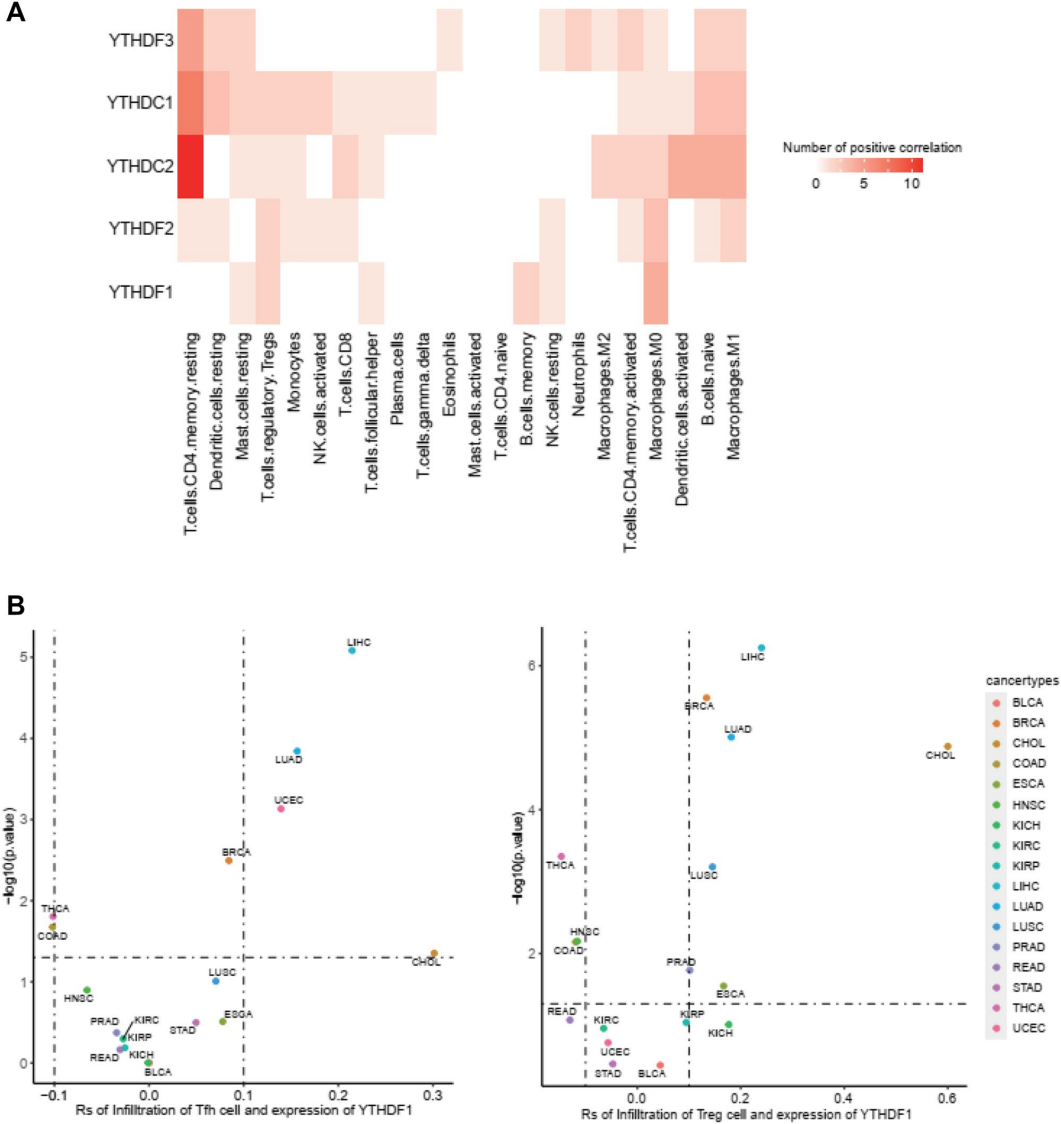

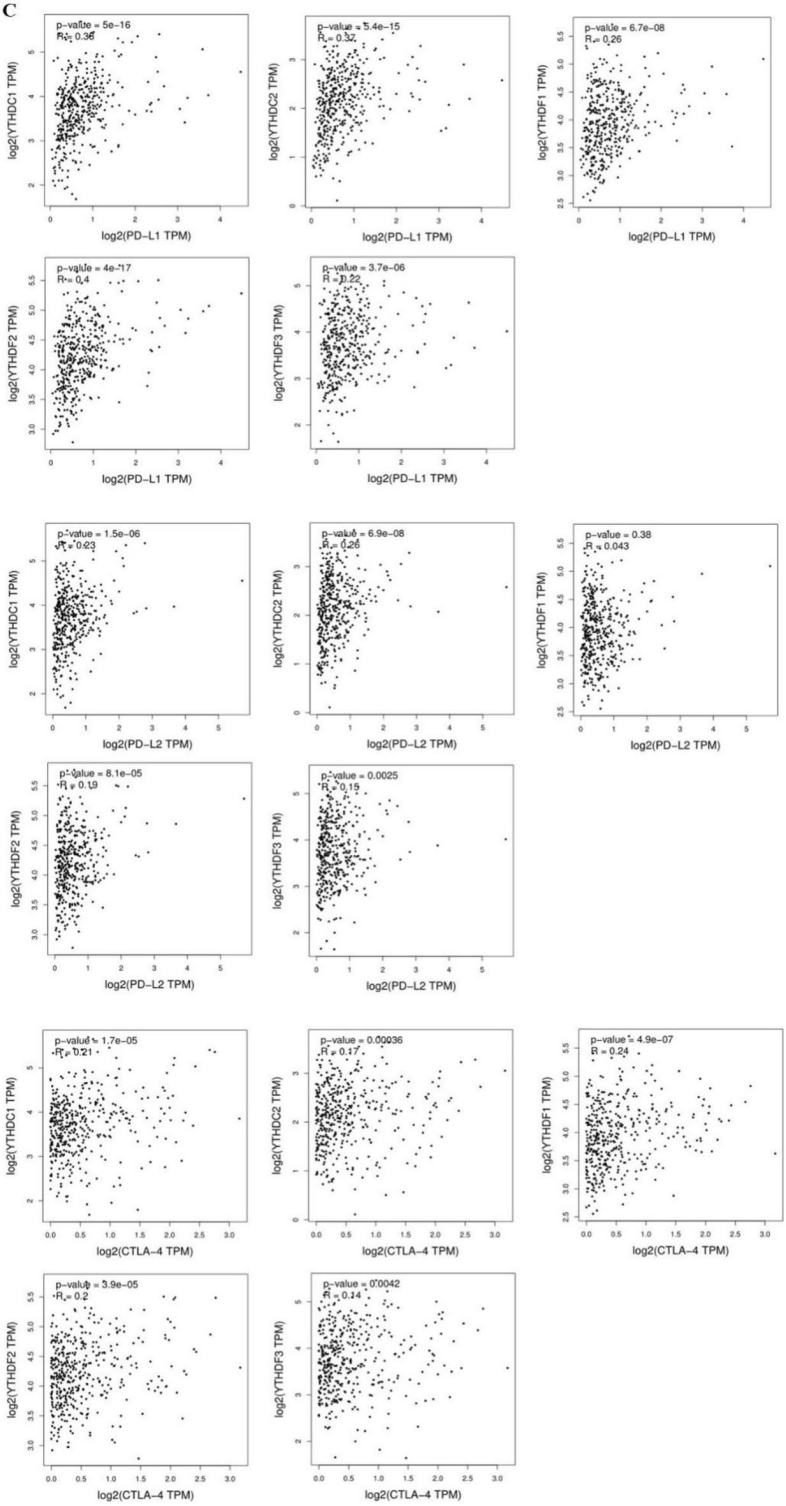
The correlation of immune cell infiltration and the expression of YTH domain family. (**A**) Heatmap color indicates the number of cancer types associated with immune cell infiltration associated with YTH domain family expression. (**B**) Correlation analysis between T_fh_, Treg cell infiltration and YTHDF1 was calculated by R. (**C**) The relationship between the YTHDF1 expression and immune marker sets in LIHC.

To explore the relationship between the immune cell infiltration in TME and YTH family genes, CIBERSORT algorithm was used to calculate the abundance of immune cell infiltration in 17 TCGA tumor samples. In addition, the relationship between the abundance of immune cell infiltration and the expression of YTH domain family genes was statistically analyzed. It was found that the expression of YTH domain family genes was associated with certain immune cell infiltration in a variety of tumors (Fig. 5A). YTH domain family sowed a positive correlation with M0 (inactive macrophages) and M1 (polarized, a pro-inflammatory type) macrophage, CD4 + T memory cell, T_fh_ cell (follicular helper T cell, a subset of CD4 + T cells) and Treg cell (regulatory T cell) (Fig. S4). YTHDC1/2 and YTHDF3 showed a close positive correlation with CD4 + T resting.

We further testified the YTH domain family expression and T_fh_ cell and T_reg_ cell and found that of T_fh_ cell infiltration was significantly positively correlated with the expression of YTHDF1 gene in LIHC, LUAD, UCEC and CHOL. There was a positive correlation between YTHDF1 gene expression and Treg cell infiltration in LIHC, CHOL, BRCA, LUAD LUSC, PRAD and ESCA (Fig. 5B). We continued to explore the relationship between the YTH domain family expression and the immune checkpoint in LIHC, PRAD and CHOL, and found that the expressions of PD-L1, PD-L2 and CTLA-4 were strongly correlated (Fig. 5C).

### The target-therapeutic prediction of YTH family

**Figure 6:**
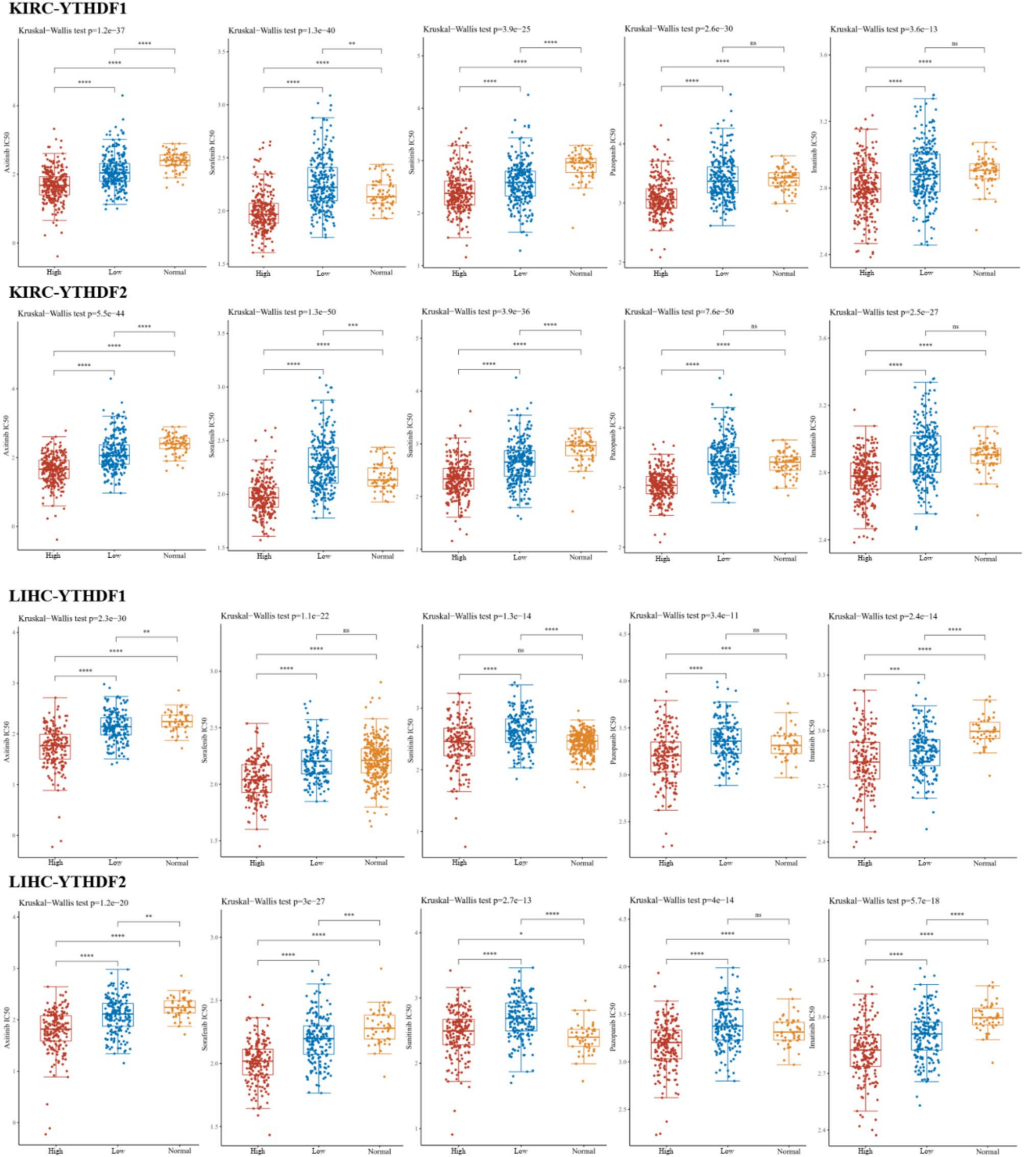
The RTK inhibitor sensitivity in LIHC and KIRC based on YTHDF1 and YTHDF2 expression. By the GDSC database, the Kruskal–Wallis test was performed among the high group, the low group and the normal group. (*p < 0.05, **p < 0.01, ***p < 0.001, asterisks (*) stand for significance levels).

Since the enrichment analysis demonstrated the YTH family had upregulated the RTK pathway. To evaluating the pharmacy response of the YTH family in liver cancer and renal cancer, we compared the RNA-sequencing expression in TCGA of YTHDF1 and YTHDF2 to the target-therapeutic response based on the largest publicly available pharmacogenomics database [the Genomics of Drug Sensitivity in Cancer (GDSC), https://www.cancerrxgene.org/]. The target medicine included Axitinib, Imatinib, Pazopanib, Sorafenib and Sunitinib. By conducting the ridge regression, the sample of KIRC and LIHC that had high YTHDF1 and YTHDF2 expression had lower IC50 of the drug density and more sensitive to the target therapy (p < 0.001) (Fig. 6).

### The clinical YTH family model for predicting the prognosis in liver cancer

**Figure 7:**
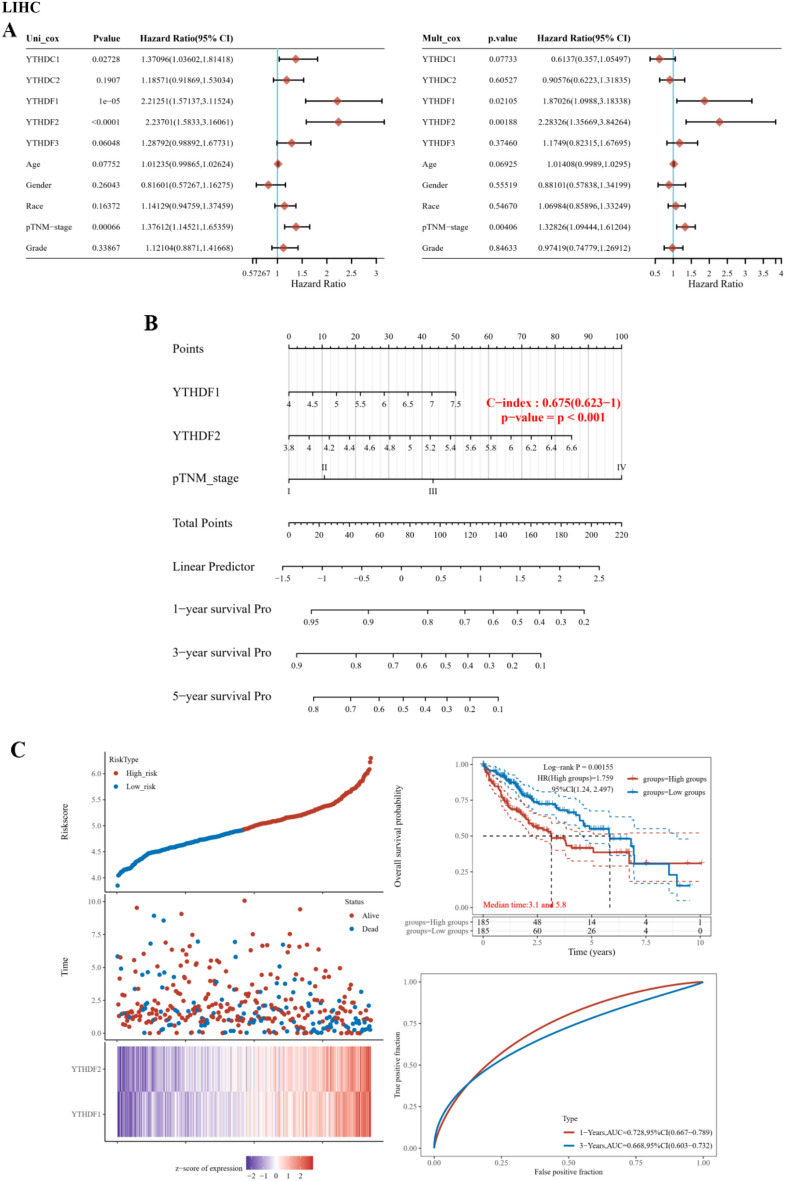
The prognosis of YTH family in liver cancer. (**A**) Univariate and multivariate Cox regression analyses were performed in liver cancer. (**B**) the nomogram gives 1-, 2- and 3-year overall survival in liver cancer. (**C**) the Riskscore, survival time and survival status of liver cancer dataset are displayed. The top scatteplot was ranked by riskscore from low to high. The middle plot distributed by the survival status and survival time. The bottom heatmap represents the YTHDF1 and YTHDF2 expression. The risk model was established and KM survival analysis between the high group and low group was performed. The ROC curves of 1- and 3-year are shown.

We estimated the risk coefficient (HR) of YTH family with age, gender, race, TNM stage and tumor grade by univariate and multivariate Cox regression in LIHC. The results demonstrated that YTHDF1(HR = 1.87, p = 0.021) and YTHDF2 (HR = 2.28, p = 0.001) were two most important biomarkers in YTH family (Fig. 7A). In addition, we used the nomogram to predict 1-, 2- and 3-year OS in liver cancer (C-index = 0.675, p < 0.001) (Fig. 7B). We also constructed a score model including YTHDF1 and YTHDF2 in LIHC, and divided the liver cancer patients into a high-score group and a low-score group. The median time of the two groups was 3.1 years and 5.8 years respectively and a high score of model was risk factor in liver cancer. The AUC of 1- and 3-year OS ROC curves was 0.728 and 0.668 respectively (Fig. 7C).

## Discussion

RNA modification has become the focus of research over past decades. With improvements and advances in RNA-immunoprecipitation techniques, multiple modification sites have been discovered, including m5C, m3C, m7G, pseudouracil and Nm modification^14–19^. Adenine was detected as the most heavily modified nucleotide, and methylation at the sixth nitrogen atom of RNA was identified as the heaviest modification^20^. N6-methyladenosine modification is the most frequent and vital methylation among the mRNAs by regulating the pre-mRNA splicing, mRNA stabilization and protein translation.

Since YT521-b, presenting as YT homology domain in eukaryotic genomes, was found as a conserved region of binding RNA-splicing protein^20^. The YTH domain family was classified into three classes: YTHDF1-3, YTHDC1, and YTHDC2. The YTHDF1 can hyperactivate cancer cell proliferation and tumor progression in colon and gastric cancers^5,21^. What’s more, the resistance against chemotherapeutics could be activated by including oxaliplatin and fluorouracil. YTHDF2 has proved to induce cancer cell proliferation and oncogenesis in leukemia and lung cancer^22,23^. It was reported that combination of YTHDF1, YTHDF2 and YTHDF3 could degrade mRNA by modifying m6A^24–26^. YTHDC1 was found to participate in splicing, degradation and mRNA transportation by binding with pre-mRNA and mRNA^27–29^. YTHDC2 could bind mRNA and elevate the efficiency of translation^30–32^. To better understand YTH domain family, we compared the correlation between the clinical phenotype, immunity, and prognosis in pan-cancer.

Analysis of the genetic expression of YTH domain family in 17 TCGA cancer types demonstrated that the distribution of YTH domain family ranged variously. M6A modification of mRNA has been reported as a double-edge sword and both the decrease and increase of m6A modification could affect the tumor progress and prognosis^33–35^. Taking YTHDF1 for example, it seems a risk factor in almost all cancers, compared with para-cancer. YTHDF1 was upregulated in liver cancer and this upregulated expression was associated with cancer development and progression, and therefore it was recognized as an oncogene. When it turns to KIRC, the YTH domain family prevented cancer development and progression. We chose LIHC to generate a model for YTHDF1 and YTHDF2 predicting the prognosis of 1- and 3-year OS. The result indicated that YTH family, especially YTHDF1 and YTHDF2, had the potential of asking as a tumor progressing biomarker.

Calculation of the relevance of the YTH family and signal pathway suggested that multiple signaling pathways were involved in including cell cycle arrest, apoptosis, wnt signaling, myc signaling and notch signaling. For example, we further investigated the relevance in specific target gene expression with YTH family, including JAG, NOTCH3, DLL in notch signaling, and twist, snail, zeb in EMT. In LIHC, both JAG1/2 and NOTCH3 showed a strong positive correlation with YTH family. Besides, zeb1/2 showed a strong correlation with YTH protein. Previous studies showed that notching signaling was associated with the control cell as oncogene or suppressor^36^. The dysregulated notch signaling affected the downstream including apoptosis, promotion of resistance, EMT, promotion of metastasis and induction of angiogenesis^37,38^. The result of pharmacy sensitivity in LIHC and KIRC bring a possibility of prediction for YTH family as a target-therapeutic biomarker that the high expression sample might be sensitive to the RTK inhibitor.

The tumor immune microenvironment has significant impact on oncogenesis, metastasis and tolerance of therapy. The immunophenotyping of the para-tumor environment also gathered for guiding the potential precising target therapy. By evaluating the immune related score between YTH family and multiple immune cell types, we discovered that CD4^+^T and macrophage were positively influenced by YTH protein. We took one step on T follicular helper cell and T regulatory cell and found that T cells also ranged variously depending on the tumor, probably acting as a double-edged sword in TME. In breast cancer, T_fh_ cell was identified as a key target of PD-1/PD-L1 blockade for immune therapy^39^. It was previously reported that T follicular helper cell was a double-edged sword, and T_fh_ help B cells generated antibodies by or dysregulating B cell by increasing the expression of PD-1^40^. On the other hand, Treg cell in TME was associated with adverse outcomes in several cancers which might be related to high-level coinhibitory molecule PD-1 expression^41–44^. According to the above-mentioned results, we concluded that YTH domain family has a strong association with immune cells of T_fh_ and Treg, though the mechanism remains to be further explored.

Our research gives a landscape of YTH domain family in different cancer types and demonstrate that YTH domain family plays distinct effects in different cancer types. With all the results based on public data, the result and underlying mechanism of YTH domain family need further experimental study.

## Conclusion

YTH domain family plays a vital effect in m6A modification in different cancers, presenting various protein expressions. However, the family is capable of influencing multiple signaling pathways and has potential of detecting the specific tumor as an oncogene or an anti-oncogene. Given the findings herein presented showing that YTHDC1 plays a protective role in COAD, BRCA, KIRP and KIRC, YTHDC2 promotes PFS in KIRC, CHOL and READ, while YTHDF3 could be a potentially protective factor in KIRC and LUSC, the certain protein of YTH domain family would be developed as a new prognosis biomarker or treatment target for cancer.

### Supplementary Information

Supplementary Figures.

## Data Availability

The datasets used and/or analysed during the current study available from the corresponding author on reasonable request.
