# Supplementary material for: Bioinformatic analysis of m6A “reader” YTH family in pan-cancer as a clinical prognosis biomarker
Source: Sci Rep. 2023 Oct 13;13:17350. doi: 10.1038/s41598-023-44143-1 (PMC10575994; doi:10.1038/s41598-023-44143-1)
Supplement: Supplementary file 1 — Supplementary Figures. [file 41598_2023_44143_MOESM1_ESM.docx]

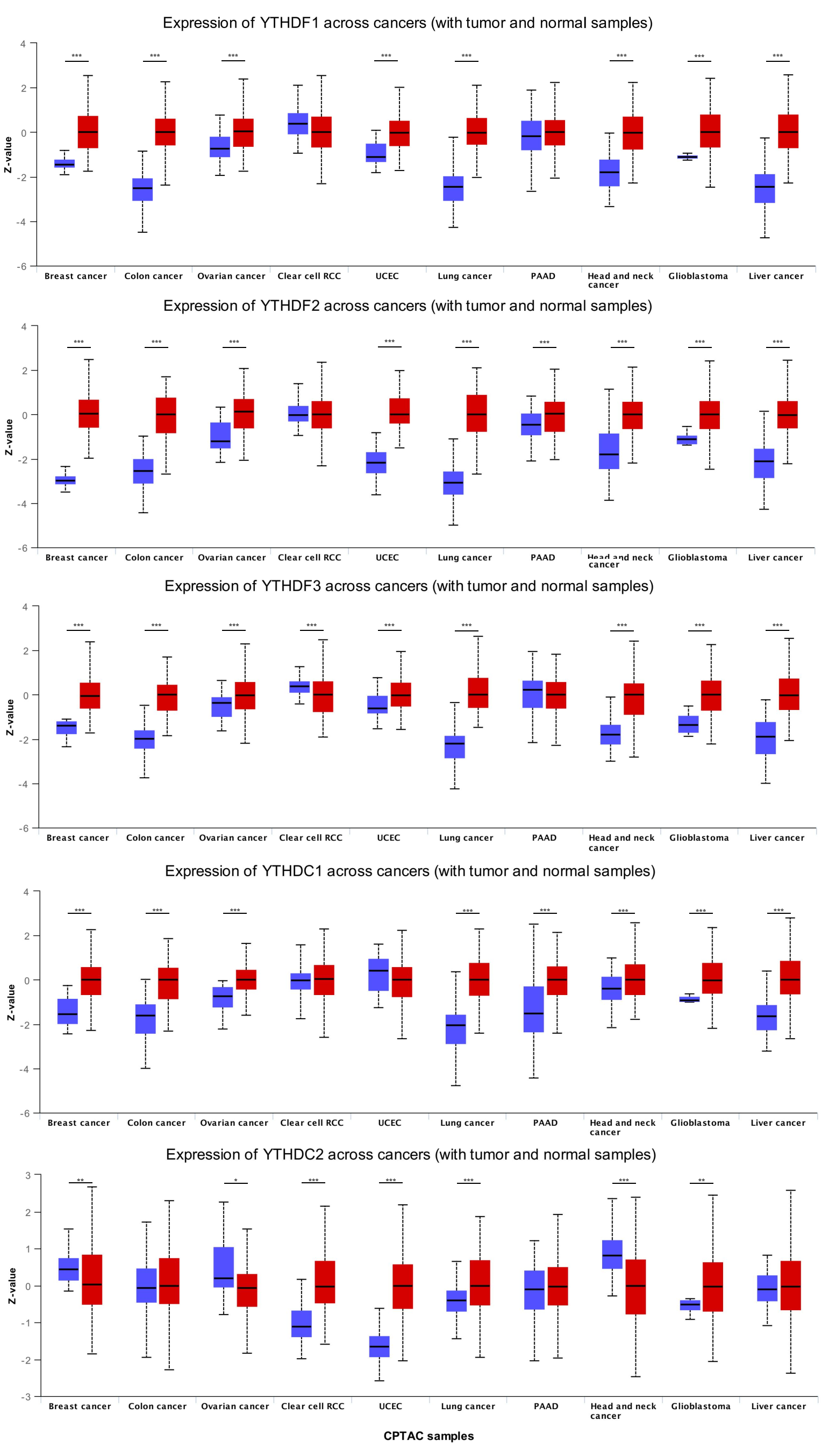


**Figure S1. The differential expression in protein levels of YTH family in pan-cancer.**

Protein expression of YTHDC1, YTHDF1, YTHDF2 and YTHDF3 is shown in different cancer types, including breast cancer, colon cancer, ovarian cancer, clear cell RCC, UCEC, lung cancer, PADD, head and neck cancer, glioblastoma, and liver cancer. ** indicates *p* value < 0.01, *** indicates *p* value < 0.001.


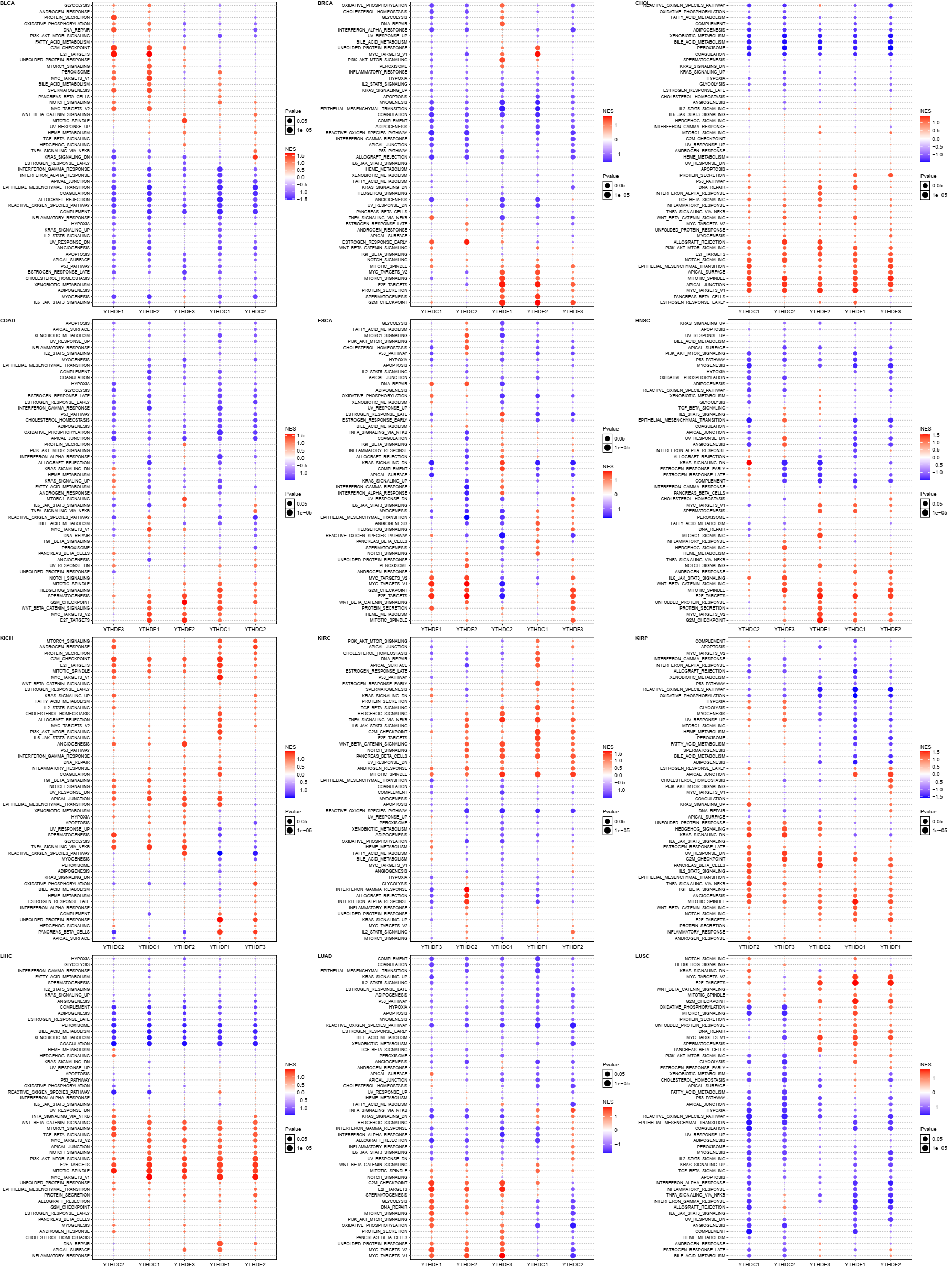


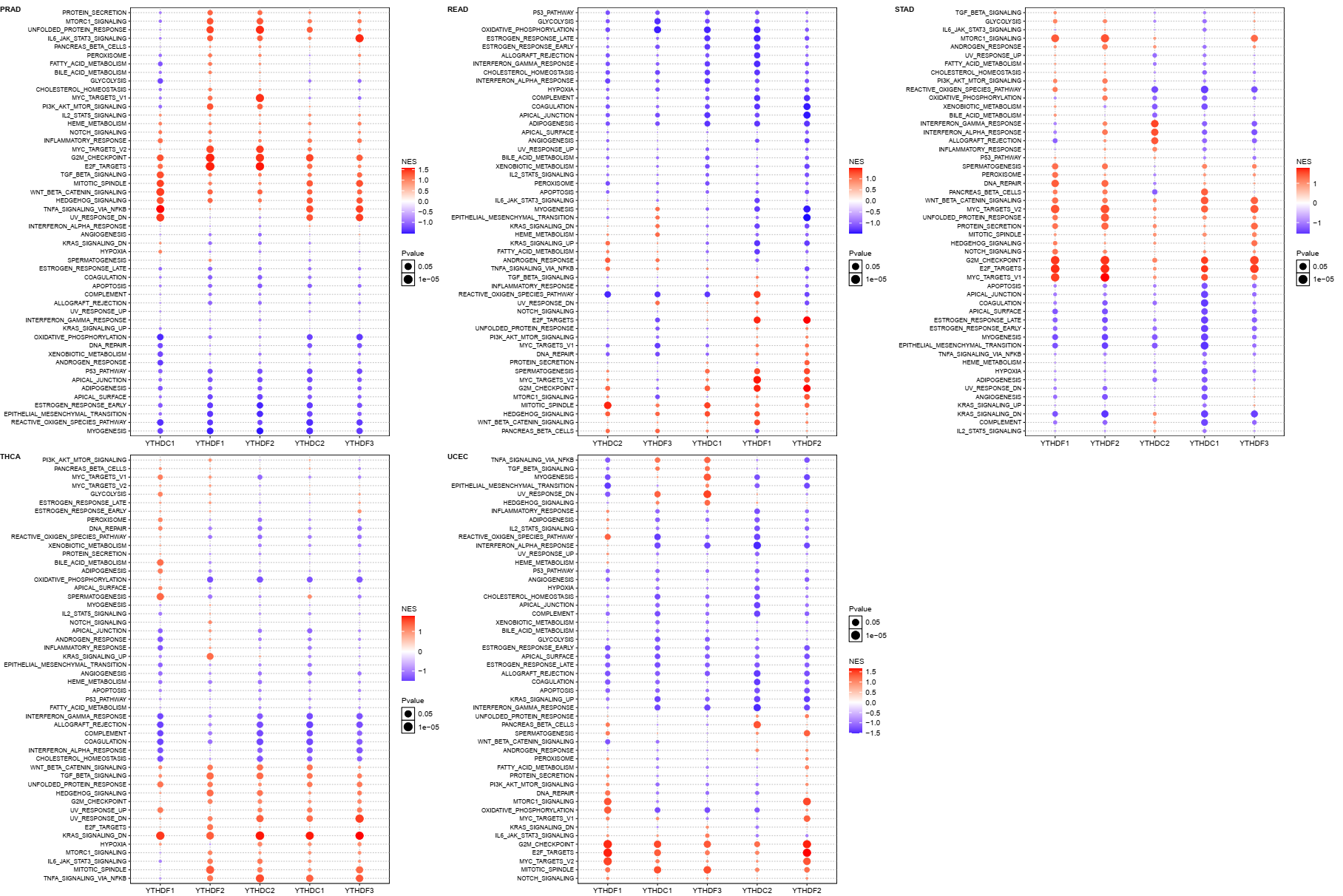


**Figure S2. GSEA enrichment analysis was performed for gene diseases significantly related to the expression of YTH family in 17 cancer types.** The expression of YTH family, including YTHDC1, YTHDC2, YTHDF1, YTHDF2, YTHDF3, is shown in different signaling pathways in 17 cancer types, including BLCA, BRCA, CHOL, COAD, ESCA, HNSC, KICH, KIRC, KIRP, LIHC, LUAD, LUSC, PRAD, READ, STAD, THCA, and UCEC.


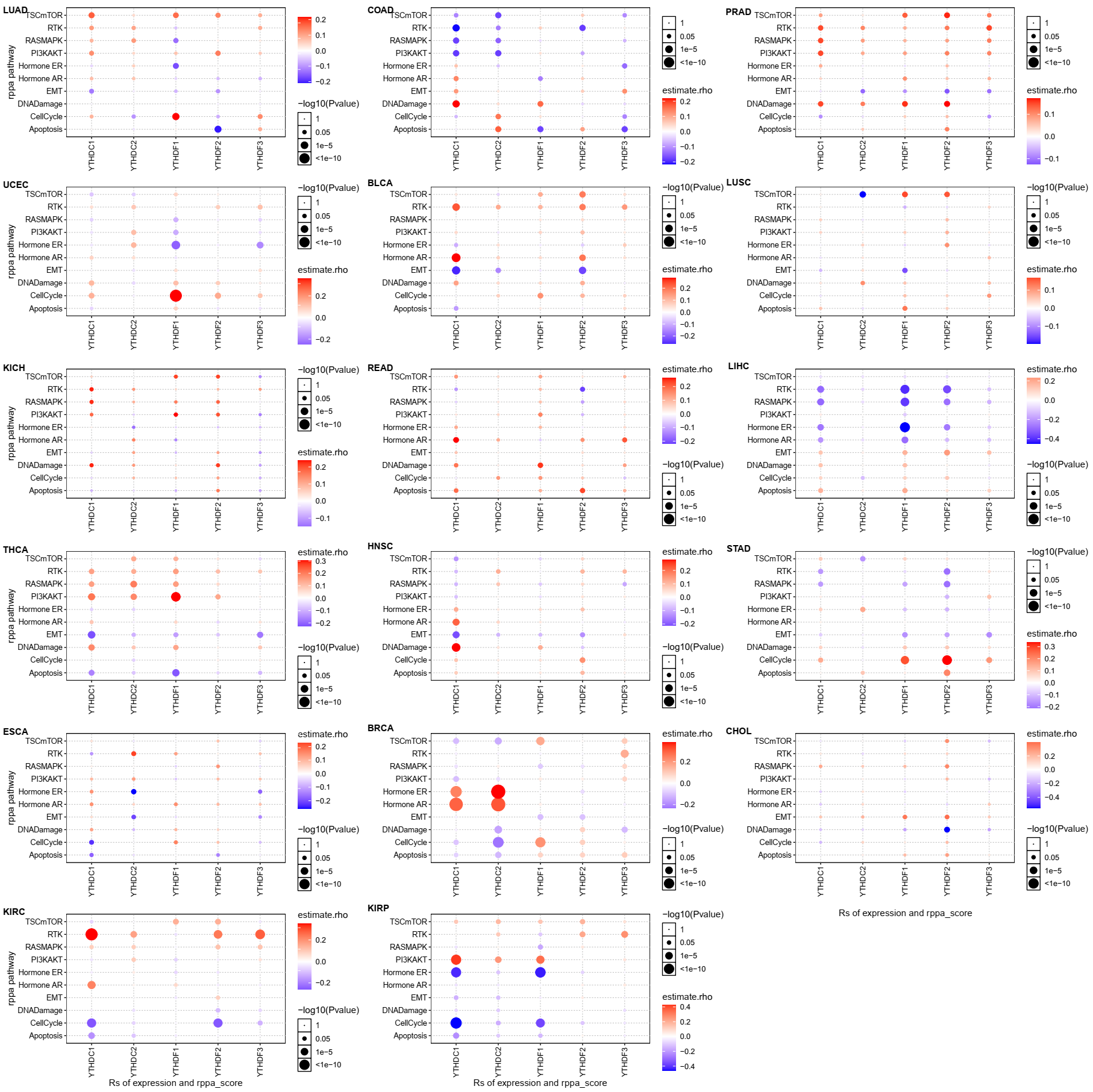


**Figure S3. The correlation between gene expression and cell signaling pathways in 17 cancer types.** The correlation between the expression of YTH family, including YTHDC1, YTHDC2, YTHDF1, YTHDF2, YTHDF3, and different signaling pathways is shown in 17 cancer types, including BLCA, BRCA, CHOL, COAD, ESCA, HNSC, KICH, KIRC, KIRP, LIHC, LUAD, LUSC, PRAD, READ, STAD, THCA, and UCEC.


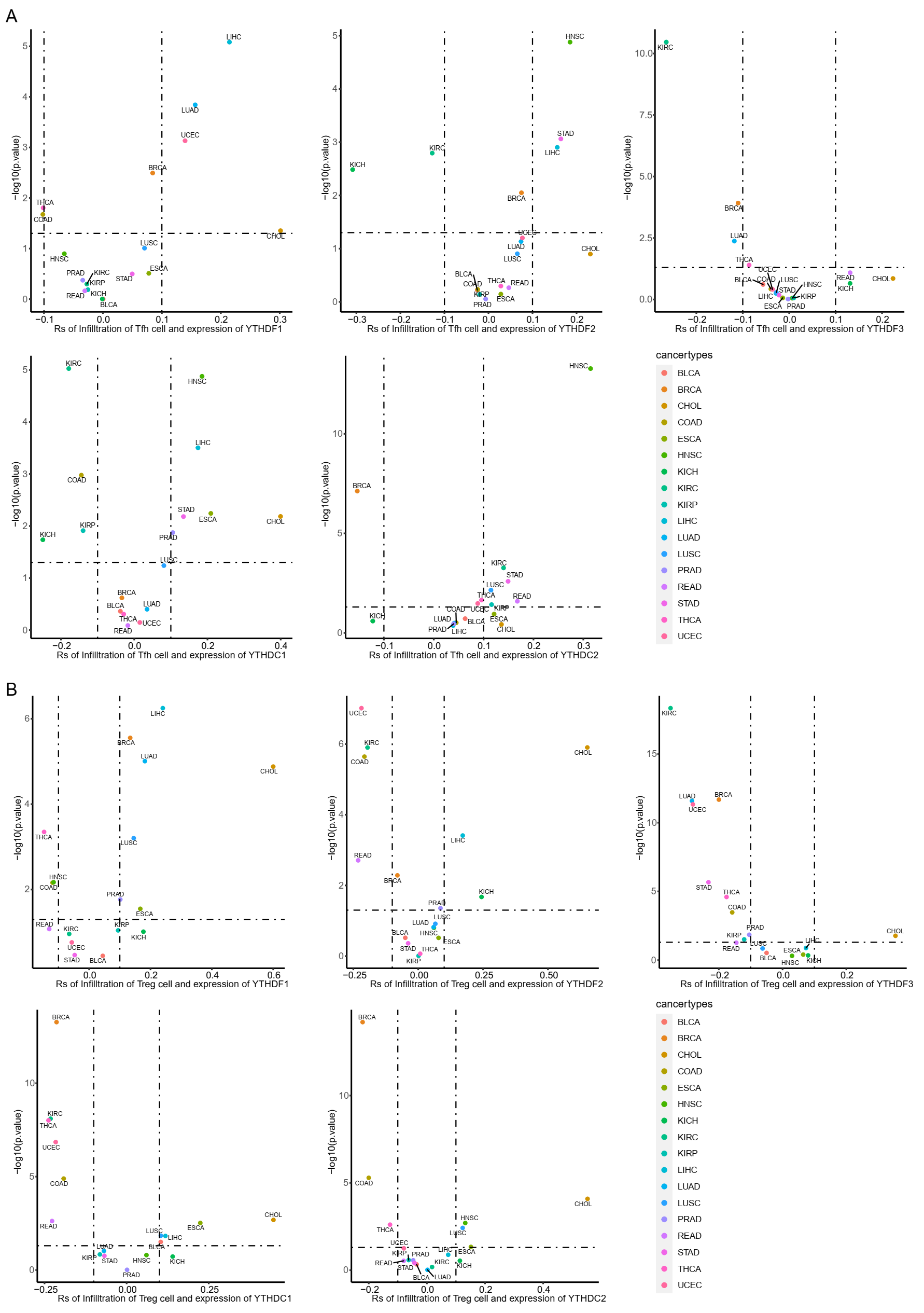


**Figure S4. The correlation between immune checkpoints and the expression of YTH family in 17 cancer types.** The expression of YTH domain family sowed a positive correlation with T_fh_ cell (**A**) and T_reg_ cell (**B**) in 17 cancer types, including BLCA, BRCA, CHOL, COAD, ESCA, HNSC, KICH, KIRC, KIRP, LIHC, LUAD, LUSC, PRAD, READ, STAD, THCA, and UCEC.
